# Post-operative emergence of acute brachial neuritis following posterior cervical laminectomy with fusion: A case report and review of the literature

**DOI:** 10.1016/j.ijscr.2019.07.065

**Published:** 2019-07-25

**Authors:** Raj H. Patel, Rishi N. Sheth

**Affiliations:** a University of South Florida, Tampa, FL, United States; b Department of Clinical Biomedical Sciences, Florida Atlantic University, Boca Raton, FL, United States

**Keywords:** Brachial neuritis, Post-surgical, Neuropathy, Shoulder pain, Neuralgic amyotrophy, Cervical laminectomy

## Abstract

**Introduction:**

Idiopathic brachial plexus neuritis or neuralgic amyotrophy is a rare neurological condition whose true etiology currently remains unknown. Epidemiologically, the incidence of this condition is exceptionally rare with only 1.6 cases for every 100,000 people (Turner and Parsonage, 1987). Symptoms present an initial acute and sudden pain to the shoulder girdle and upper arm which is followed by a sense of profound weakness and numbness to the upper arm (Parsonage and Turner, 1948). Localized neuropathy within the arm-pit region may also be presented. The pain often exacerbates upon movement of the shoulder. Due to the anatomic location affected and the nature of the clinical symptoms presented, accurate diagnosis of brachial plexus neuritis poses a challenging diagnostic task for physicians due to remarkably similar symptoms expressed by differential diagnoses.

**Presentation of case:**

Here, we report the case of a 55-year-old woman who underwent surgery entailing cervical laminectomy with instrumented fusion. She presented with postoperative symptoms of severe pain in the left arm with significant weakness within 24 h after surgery. A diagnosis of brachial plexus neuritis was made based on the symptoms presented and upon review of imaging scans.

**Discussion:**

After a six-month follow-up visit, the patient recovered from the brachial neuritis but has residual numbness in the hand. The presentation of this case serves to transmit three fundamental purposes. First, this case serves to establish an intriguing possible association of the post-surgical period of cervical laminectomy with acute brachial neuritis and signifies the importance post-operative linkage with brachial neuritis in general. Second, this case also highlights the importance of close clinical monitoring of patients with unique symptoms within the postoperative follow-up period to ensure successful improvement and accurate diagnosis.

**Conclusion:**

As an underdiagnosed and relatively obscure condition, this case serves as an imperative reference for physicians to illuminate differential diagnosis of similar symptomatic conditions and also to promote knowledge of brachial plexus neuritis which can lead to an early and precise diagnosis.

## 1 Introduction

Brachial neuritis has various designations such as parsonage-turner syndrome, neuralgia amyotrophy. Brachial neuritis is a form of peripheral neuropathy of the brachial plexus. The condition was originally described by Drs. Parsonage and Turner after they began to see an abnormal pattern of cases during army work for the United Kingdom and India Command [10]. Although this rare neurological condition has an unclear true etiology, there have been previous reports that show possible preceding factors such as vaccinations, autoimmune infections, genetic predispositions, trauma at a remote site, or pregnancy [16,14,11]. Clinical symptoms include abrupt and acute onset of pain within the shoulder girdle and upper arm followed by numbness and weakness within the upper arm [10]. Symptoms are usually unilateral and patients report a distinct sharp and radiating pain circulating within the arm-pit region. Onset of sharp and excruciating pain denotes the beginning of symptoms which then later transitions to weakness and numbness over the course of a few hours to days. The severe initial pain usually subsides after weakness or paralysis develops. This incidence and observation of pain translating to weakness is crucial to the accurate diagnosis of acute brachial plexus neuritis. Brachial neuritis is more prevalent in men between the ages of 30–70, and is commonly diagnosed on the right side [1]. Prognosis of brachial neuritis is optimistic, with most individuals recovering 70–90% of their original function after three years [12]. Treatment options are limited but include including oral steroids to reduce inflammation, narcotic or neurotropic medications for severe pain, and physical therapy. Surgical intervention is rare but may be considered if the individual has not improved after two-years of clinical treatment and may involve nerve grafting or tendon transfer. We report a case of post-operative emergence of brachial plexus neuritis which developed after the patient underwent surgical procedure for cervical stenosis. Our work has been reported in line with the SCARE criteria [20].

## 2 Case report

### 2.1 History

A 55-year-old woman presented with neck pain for many years since a motor vehicle accident twenty-one years ago. Additionally, patient presented with paresthesia and pain in the right arm with a burning sensation near the shoulder girdle. Intermittent numbness in the toes and bilaterally hands was also reported.

### 2.2 Examination

Physical examination reports revealed restricted range of motion of the neck when turning the body to the left. Long tract signs were positive for Babinski and Hoffman signs with hyperreflexia in the lower extremities and the right arm. Weakness was presented in the right wrist extensor and the right hip flexor.

**Fig. 1:**
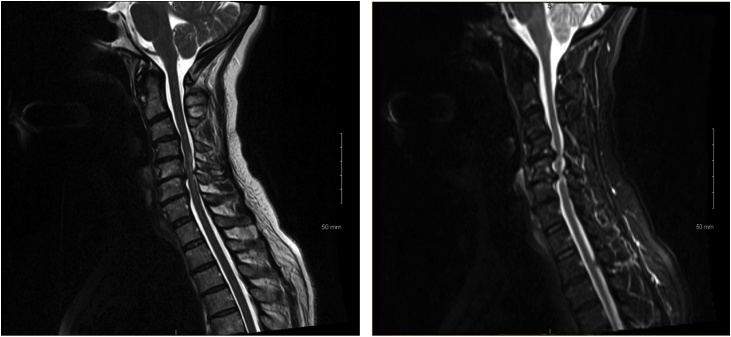
Preoperative MRI Scans showing cervical spine cord compression.

A magnetic-resonance-imaging scan (Fig. 1) showed deformity of the cervical spine with kyphotic angulation and spinal cord compression. At C5-C6, there was grade 1 retrolisthesis present and at C3-4 there is grade 1 anterolisthesis. Severe canal stenosis with cord compression and severe bilateral foraminal stenosis with abnormal signal in the spinal cord at C5-C6 and C4-C5, suggest myelomalacia. At C6-C7, there was right-sided disc herniation causing cord compression on the right side.

### 2.3 Operation

Surgical intervention included cervical laminectomy from C3 to C7 with lateral mass fusion from C3 to C7. Neurophysiological monitoring (SSEP and EMG) was stable throughout the duration of the surgical procedure, satisfactory signal response was also obtained from all four extremities before and during the surgery. No complications developed during surgery and in the recovery room the patient was conscious and neurologically unchanged as preoperative exam.

### 2.4 Postoperative course

**Fig. 2:**
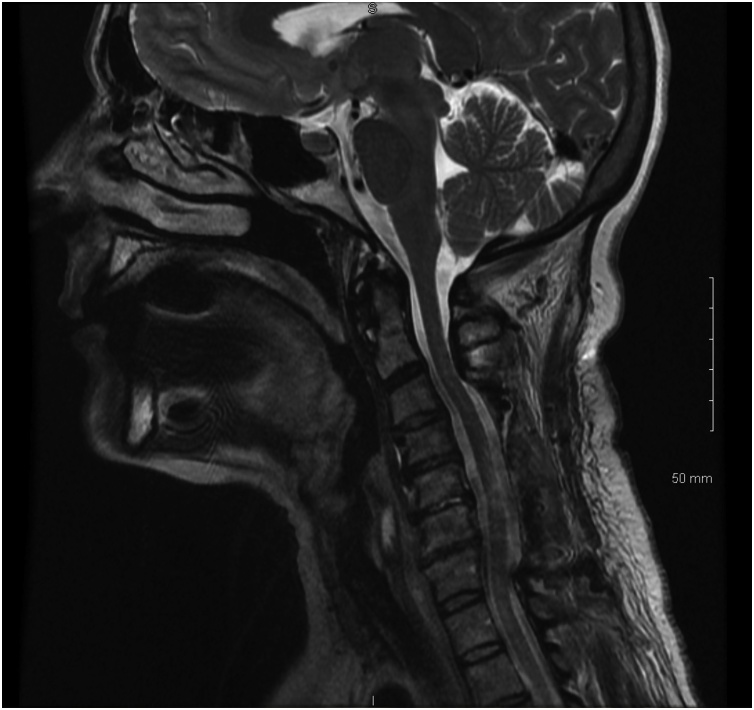
Postoperative MRI Scan of the Cervical Spine.

**Fig. 3:**
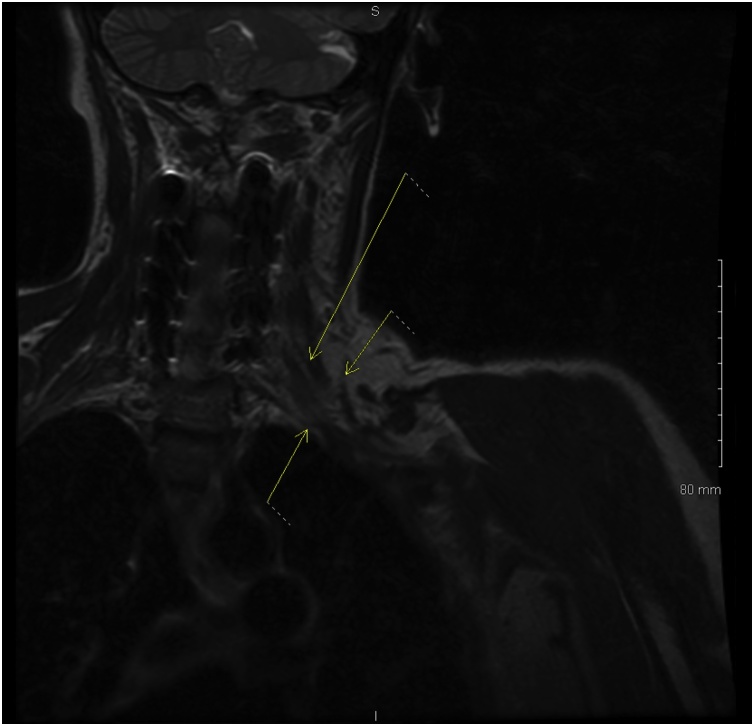
Postoperative onset of Brachial Neuritis shown through MRI.

Twenty-four hours following the surgical procedure, the patient presented with significant pain in the left para-cervical and shoulder area going into the arm. There was numbness in the entire arm in no particular dermatome. The next day she had more pain in the left arm with newly developed weakness in the deltoid, biceps, triceps, wrist extensor, grip and intrinsic muscles. The patient had difficulty using the arm both proximally and distally. An MRI was conducted post-operatively which shows the well decompressed cervical spine (Fig. 2). An MRI scan of the brain was also conducted which showed negative findings. Post-operative MRI scan of the brachial plexus revealed edema within the three trunks of the brachial plexus (Fig. 3). The diagnosis of acute brachial plexus neuritis was thus made, based on the pattern, chronology and findings of the patient’s symptoms and on the MRI scan of the brachial plexus. Physical and occupational therapy was recommended for the left arm. During a two-week follow-up postoperative visit the patient presented increased strength in the left arm as an indication of noteworthy progress. In a later two-month follow-up visit, the symptoms improved and strength in the left arm also improved by 80%. The patient had complaints of residual pain within the posterior neck region and upper trapezius area. In a final six-month follow-up visit, the patient presented occasional neck pain, but very good range of motion of the neck. The strength and numbness in the left arm had recovered more, however there was still residual numbness in the thumb, index and the middle finger on the left hand. The patient demonstrated normal coordination and motor strength. Bilateral arm strength was also significantly improved with a 5/5 rate of strength. After six-months of efficient physical therapy and treatment, the patient has presented significant postoperative improvement and has displayed complete recovery from the brachial neuritis.

## 3 Discussion

Acute brachial neuritis is a rare and under-recognized diagnosis for post-operative cervical pain. Etiology of brachial neuritis is currently unknown, however, previous medical literature suggests that possible influences could be auto-immune, post-vaccination, post-surgical or genetic [14,9,17,5,18]. To the best of our knowledge, reports of post-surgical brachial neuritis have been very scarce in the medical literature and with only two relating to cervical decompression surgery [9,17,5]. Previous medical literature which has documented post-operative brachial neuritis differs significantly from this case in numerous facets. For example, past reports show significantly delayed onset of BN symptoms in which patients present with pain a few weeks after surgery [9,17]. Also, variance in the site of surgical intervention is also a component of divergence when comparing past reports [5].

Although a link to the site of surgery has not been established, this report allows for a greater illumination to display a possible correlation between site of surgery and BN. Post-surgical brachial neuritis is typically seen to emerge several weeks to months following surgery, however, in this documentation the onset of brachial neuritis was observed within 24 h of surgery with the site of surgery being dominantly within the cervical area [6]. This is quite unique and imperative, as such a rapid emergence has not been documented in the past. It demonstrates that the onset of brachial neuritis is highly variable and could present anytime post-operatively. Previous reports have also focused on discussing C5 palsy as a differential diagnosis due to site of the surgery, however, postoperative C5 palsy was eliminated in our report due to the patient’s presentation of non-dermatomal distribution of pain and weakness in the right arm. Also, this involved dissimilar roots and patient motor exam was intact directly after surgery [9].

The novelty of this report rests partly in the premise of the distinctive timing and potency of the symptoms presented. The literature indicates a former report where a young boy was diagnosed with brachial neuritis 72 h post-operatively, following subcutaneous mastectomy [19]. This previous report clarifies the importance of accurate and timely diagnosis, yet also differs from this report in several areas. The case presented herein involves an unusually rapid onset of BN symptoms, where diagnosis was made within 24 h of surgery. Furthermore, this previous report involved a significantly delayed and ambiguous presentation of symptoms which was diagnosed as a cervical spine injury from hyperextension of the neck during intubation, whereas the symptoms presented by the patient in this report showed a clear and definite diagnosis of BN post-operatively. The site of surgery is also dissimilar in both of these reports, as this case involved a posterior cervical laminectomy with fusion.

Accurate and timely diagnosis of brachial neuritis can become a challenging task for physicians. There are several other similar conditions including ulnar nerve neuropathy, cervical radiculopathy, adhesive capsulitis, shoulder arthritis, and calcifying tendonitis [15,18]. It is important to conduct an accurate diagnosis as the treatment plan for brachial neuritis differs significantly amongst other differential diagnoses. Electromyogram and nerve-conduction studies remain as widely chosen routes to accurately localize and define parameters and location of the plexopathy [3,15]. These studies were considered for the patient, however, the patient decided against it as they were supplementary. It is important to recognize differential diagnosis as it is very easy to inadvertently misdiagnose acute brachial neuritis due to severe shoulder pain being a primary symptom in various other conditions. Brachial neuritis however, has a marked distinction in that the initial severe pain later translates to weakness and paresthesia after a few hours to days, which is not seen in other differential diagnoses [7].

Surgical trauma has been well-documented influence for post-surgical complications and could be attributed to influencing factors for such cases [4]. However, in our case, there is a strong likelihood and case that this was not a major impact based on the pattern, location, nature, and severity of the symptoms. The patient had an unchanged neurological examination result which was conducted directly after surgery in the recovery room. In addition, neuro-monitoring was stable throughout the surgery. It is clear that surgical stress did not have an influence on the patient’s onset and development of brachial neuritis. In general cases where BN has been induced via surgical stress or intraoperative positioning, neurophysiological monitoring has been unstable during the surgery. In our case, the patient’s neurophysiological monitoring was stable as were EMG and SSEP findings. However, it can be argued that surgical stress could have had a triggering effect in the development of subsequent symptoms later on in the course of post-operative recovery. In other words, a possible inference that the traction or positioning during surgery could have influenced the development of the syndrome can be speculated. Central development of the symptoms in the patient however cannot be attributed solely to surgical stress or auto-immune response, but may be argued to be of systematic and triggering reaction from the combination of these two influences as has been speculated in previous cases.

A review of the medical literature indicates that post-operative brachial neuritis may be triggered by an auto-immune influence after response of the immune system to a virus in the nerve roots, although these speculations are unsubstantiated [2]. Furthermore, past literature shows that certain post-surgical neuropathies can be attributed to inflammatory causes and mechanical factors such as contusion or compression as well as influencing factors being through spinal anesthesia [4,13]. However, in the case presented, the patient presented well in the recovery room, and the onset of the brachial neuritis was acute which is a significant defining factor of the discrepancy between this case and other post-operative brachial neuritis cases.

Brachial neuritis typically resolves fairly quickly in patients, and full range of motion with strength is usually returns within the course of a year [8]. Initial treatment typically places a focus on pain management and utilization of analgesics to control the patient’s pain which can often be very sharp and severe onset. After the pain translates into weakness, rehabilitative exercise or physical therapy should be prescribed and recommended for the patient to maintain strength in the arm and regain range of motion. It is imperative to encourage and support the patient in that the condition improves but can be slow-paced. If the C5 area has been profoundly impacted, a sling can also be recommended to avoid subluxation of the humerus [8]. Common modes of treatment may include oral steroids to reduce inflammation, rest, narcotic medications, or extensive neuropathic pain management. In this case, the patient was given oral steroids, muscle relaxants and narcotic medications which were moderately beneficial in alleviating the severe and sharp pain. Prognosis of brachial neuritis is generally good with accurate and timely diagnosis and treatment [12].

## 4 Conclusion

The patient described herein represents a unique case, and to the best of our knowledge, the first documentation of postoperative onset of brachial neuritis within 24 h of cervical decompression surgery. This case is significantly distinct from other cases of post-operative emergence of brachial neuritis as common differential diagnosis were ruled out early in the process due to the highly distinction of BN symptoms. Diagnosis was confirmed clinically and radiologically. Furthermore, it is imperative that the surgeon regards possible rare diagnosis in evaluating post-operative neurological deficits. Overall, the patient responded quite well to aggressive physical therapy to the left arm, successfully reversing the deficits of the symptoms within six-months. Recovery and prognosis of brachial neuritis is generally effective, but can often be slow-paced.

## Sources of funding

None.

## Ethical approval

All procedures/reporting has been done in ethical accordance. Ethical approval from the institution is not necessary in our particular case as our study is exempt.

## Consent

Patient consent has been obtained, identifying details have been omitted from the case report.

## Author contribution

1st Author: Wrote the majority of the paper.

2nd Author: Performed surgery, reviewed/edited the discussion and case report components.

## Registration of research studies

N/A.

## Guarantor

Both authors accept full responsibility for the work and/or the conduct of the study, have access to the data, and controlled the decision to publish.

## Provenance and peer review

Not commissioned, externally peer-reviewed.

## Declaration of Competing Interest

None.
